# How to Prevent Rubella Epidemics and Congenital Rubella Syndrome: Lessons From 42 Years of Longitudinal Epidemiology in Osaka Prefecture, Japan (1982–2023)

**DOI:** 10.1093/infdis/jiae402

**Published:** 2024-08-14

**Authors:** Daiki Kanbayashi, Takako Kurata, Yuko Kaida, Tatsuya Miyoshi, Fumika Okayama, Tetsuo Kase, Jun Komano, Kazuo Takahashi, Kazuyoshi Ikuta, Kazushi Motomura

**Affiliations:** Division of Microbiology, Virology Section, Osaka Institute of Public Health, Osaka-shi; Division of Microbiology, Virology Section, Osaka Institute of Public Health, Osaka-shi; Laboratory Section, Fujiidera Public Health Center of Osaka Prefectural Government, Fujiidera-shi; Sakai City Institute of Public Health, Sakai-shi; Sakai City Institute of Public Health, Sakai-shi; Department of Public Health, Osaka Metropolitan University Graduate School of Medicine, Osaka-shi; Department of Microbiology and Infection Control, Faculty of Pharmacy, Osaka Medical and Pharmaceutical University, Takatsuki-shi; International University of Health and Welfare, Otawara-shi; Division of Microbiology, Virology Section, Osaka Institute of Public Health, Osaka-shi; Research Institute for Microbial Diseases, Osaka University, Suita-shi; Division of Public Health, Osaka Institute of Public Health, Osaka-shi, Japan

**Keywords:** congenital rubella syndrome, immunization program, rubella, rubella-containing vaccine, surveillance

## Abstract

**Background:**

Despite the introduction of rubella-containing vaccine into routine immunization in 1977, rubella has not been eliminated in Japan. This study aimed to validate the immunization strategy and highlight crucial elements of the elimination program.

**Methods:**

We scrutinized cases of rubella and congenital rubella syndrome (CRS). Additionally, we analyzed the national vaccination coverage, seroprevalence, and number of maternal rubella-related spontaneous or artificial fetal deaths.

**Results:**

The shift from selective to universal immunization significantly reduced rubella cases, coupled with increased seroprevalence in children. However, rubella resurged in 2012 to 2013 and 2018 to 2019, which was virologically and serologically confirmed to be associated with imported rubella virus and susceptible males. Although the disease burden of CRS may have been suppressed in the past by the large number of spontaneous or artificial fetal deaths, the incidence rate of CRS was comparable to that of the 1960s to 1980s. Cases of breakthrough infection and CRS were identified in females who were considered to have a history of single-dose vaccination.

**Conclusions:**

Even with universal immunization, future epidemics and severe outcomes cannot be prevented unless immunization gaps are closed. Furthermore, CRS and breakthrough infection are not completely prevented by single-dose vaccination, indicating the need for second-dose vaccination.

## Teaser

Longitudinal epidemiology of rubella has shown that future epidemics and severe outcomes cannot be prevented unless immunization gaps are closed. In addition, congenital rubella syndrome and breakthrough infection are not completely prevented by a single-dose vaccination, indicating the need for second-dose vaccination.

## Graphical Abstract

**Graphical Abstract:**
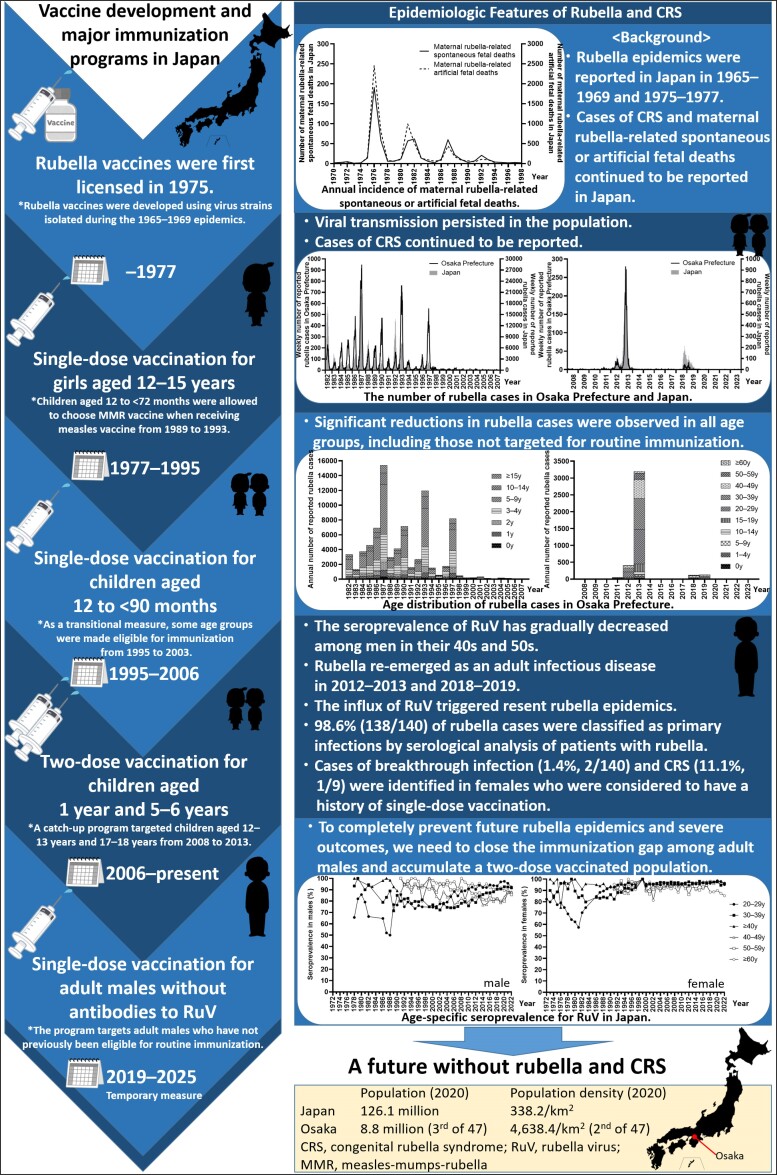
This graphical abstract is also available at Tidbit: https://tidbitapp.io/tidbits/how-to-prevent-rubella-epidemics-and-congenital-rubella-syndrome-lessons-from-42-years-of-longitudinal-epidemiology-in-osaka-prefecture-japan-1982-2023-ac75c7ed-4be2-4890-a7ab-45929a8a8f69?utm_campaign=tidbitlinkshare&utm_source=IO

Rubella is a mild contagious disease caused by the rubella virus (RuV) [1]. RuV infection in pregnant women can result in fetal death or congenital rubella syndrome (CRS) [1]. The development of rubella-containing vaccines (RCVs) has made rubella and CRS preventable [1]. By 2020, RCVs had been introduced in 173 of 194 (89%) World Health Organization member countries, with 93 (48%) achieving rubella elimination [2]. In addition, vaccine introduction equity has greatly improved in lower-income countries, and global efforts to eliminate and eradicate rubella are ongoing [2, 3].

In Japan, RCVs were developed with virus strains isolated during the 1965–1969 epidemics, and 5 strains—Matsuura, TO-336, TCRB19, Takahashi, and Matsuba—were licensed [4, 5]; the TCRB19 and Matsuba strains are currently discontinued. When RCVs were first licensed in Japan in 1975, there was no international consensus on the target groups for vaccination: (1) selective immunization of susceptible adolescent girls and women of childbearing age or (2) universal immunization targeting children. In August 1977, a single-dose rubella vaccination was introduced into the national immunization program [6]. However, until 1994, vaccination was limited primarily to adolescent girls aged 12 to 15 years, and coverage ranged from 27.3% to 74.0% of the target population [6–8]. This decision was based on several factors: (1) the government's desire to immunize as many women as possible before the next epidemic to prevent CRS, (2) concerns about the duration of vaccine-induced immunity because RCVs had just been developed, and (3) the perceived reduced risk of infection in pregnant women around the vaccine recipient because families with junior high school students were less likely to have pregnant women at home [9]. From April 1989, children aged 12 to <72 months were allowed to choose measles-mumps-rubella vaccine when receiving the measles vaccine. Yet, this vaccine was withdrawn in April 1993 because of a relatively high incidence of aseptic meningitis caused by a mumps component [10, 11]. In 1995, the target for routine immunization was changed to include children aged 12 to <90 months [6]. As a transitional measure, the vaccine was administered to elementary school students in first or second grade in fiscal year 1995, elementary school students in first grade in fiscal years 1996 to 1999, children aged 12 to 15 years (April 1995–September 2003), and individuals born between 2 April 1979 and 1 October 1987 (November 2001–September 2003), although coverage was limited to 14.7% to 55.9% of the target population [6, 7, 8]. In 2006, to address primary and secondary vaccine failure, the 1-dose vaccination schedule was changed to a 2-dose schedule for children aged 1 year and 5 to 6 years with the measles-rubella vaccine [12], and this schedule remains in place today. In addition, from April 2008 to March 2013, a catch-up program with the measles-rubella vaccine targeted children aged 12 to 13 years and 17 to 18 years [12]. Moreover, another catch-up immunization program is underway from February 2019 to March 2025 to immunize males born between 2 April 1962 and 1 April 1979, who do not have anti–RuV-specific antibodies [13].

Despite the introduction of RCVs into Japanese routine immunization in 1977, rubella elimination has not been achieved in Japan even after 46 years. The presence of susceptible adults is epidemiologically presumed to be a factor preventing elimination, although there is no study to prove this presumption by serologic analysis of patients with rubella. In this study, we analyzed epidemiologic, laboratory, and clinical data in Osaka prefecture, which represents approximately 7% of the Japanese population (8.8 million/126.1 million on 1 October 2020) [14], with the advantage of access to all materials, including clinical specimens, to validate the immunization strategy and underscore key components of the rubella control and elimination program.

## METHODS

The infectious disease surveillance system in Japan consists of 2 main components: patient reporting and pathogen reporting [15]. We collected and analyzed epidemiologic, laboratory, and clinical data based on this system.

### Epidemiologic Surveillance: Patient Reporting

#### Sentinel Surveillance of Rubella: 1982–2007

Sentinel surveillance of rubella was initiated in Japan in 1981 [15, 16]. In Osaka prefecture, 164 hospitals or clinics were selected as sites to initiate sentinel surveillance in 1982. After the establishment of the Prevention of Infectious Diseases and Medical Care for Patients With Infectious Diseases in September 1998, which took effect in April 1999, the program became a statutory initiative. Sentinel hospitals and clinics reported weekly age-specific numbers of newly diagnosed cases. Due to several changes in age categories over time, the age distribution during this period was summarized as follows: 0, 1 to 4, 5 to 9, 10 to 14, and ≥15 years.

#### Notifiable Disease Surveillance of Rubella: 2008–2023

Since January 2008, rubella surveillance in Japan has been strengthened to require reporting by all hospitals and clinics [15]. Identification of rubella cases was based on the presence of specific symptoms, including generalized erythema and/or erythematous papules, fever, and lymphadenopathy [17]. Cases were reported in 2 categories: clinical diagnosis (presence of all 3 clinical symptoms) and laboratory diagnosis (presence of at least 1 clinical symptom and a positive laboratory test result) [17]. Additionally, details such as age, sex, and clinical symptoms were recorded. In 2018, further measures to control rubella were promoted by amending the “Guidelines for the Prevention of Specific Infections: Rubella” [13]. All physicians are now required to submit a notification upon clinical diagnosis of rubella; perform serum antibody titer measurement, such as anti–RuV-specific IgM antibodies; and submit specimens, such as throat swab, blood, and urine samples, for RuV detection [13].

#### Notifiable Disease Surveillance of CRS: 1999–2023

In 1999, CRS was officially classified as an infectious disease, requiring notification of all cases [12]. Identification of CRS cases was based on the presence of 1 or more specific symptoms—including cataract, congenital glaucoma, congenital heart disease, deafness, pigmentary retinopathy, purpura, splenomegaly, microcephaly, mental retardation, meningoencephalitis, radiolucent bone lesions, and jaundice—in addition to testing positive for RuV or specific antibodies [18].

### Detection and Molecular Epidemiology of RuV: Pathogen Reporting

RuV strains are classified into 2 clades, which are further subdivided into 10 (1a, 1B–1J) and 3 (2A–2C) genotypes [1, 19]. To improve the resolution of genetic classification, a subdivision of these genotypes has been proposed [20]. Clinical specimens were collected from patients with a clinical diagnosis of rubella or measles. RuV RNA was prepared and detected, as previously described [21–24]. For nucleic acid amplification test (NAT)–positive specimens, the World Health Organization–recommended 739-nucleotide window region (positions 8731–9469) within the *E1* gene was amplified and sequenced [25]. The phylogenetic tree was constructed by the maximum likelihood method and Tamura-Nei model [26] in MEGA software [27].

### Serologic Analysis of Patients With Acute Rubella

For NAT-confirmed rubella cases, anti–RuV-specific IgG antibodies in blood samples were measured with the Virus Antibody EIA (Seiken) Rubella IgG Kit with Baylor strain as antigen (Denka). An anti–RuV-specific IgG antibody titer <4.6 IU/mL was interpreted as negative, 4.6 to 9.2 IU/mL as equivocal, and ≥9.2 IU/mL as positive. The avidity of anti–RuV-specific IgG antibody was determined by the EUROIMMUN Rubella ELISA IgG Avidity Kit with HPV-77 strain as antigen (EUROIMMUN Medizinische Labordiagnostika AG). The relative avidity index (RAI) was measured for samples that were positive or equivocal for anti–RuV-specific IgG antibodies and expressed as a percentage based on the optical density values with and without denaturant. RAI <40% was interpreted as low-avidity antibodies, 40% to 60% as equivocal, and ≥60% as high.

### Ethical Approval

This study was conducted in accordance with the relevant laws and was approved by the Ethics Committee of the Osaka Institute of Public Health (approval 1302-06-7).

## RESULTS

### Epidemiologic Surveillance

#### Epidemiologic Trends of Rubella and CRS

**Figure 1.**
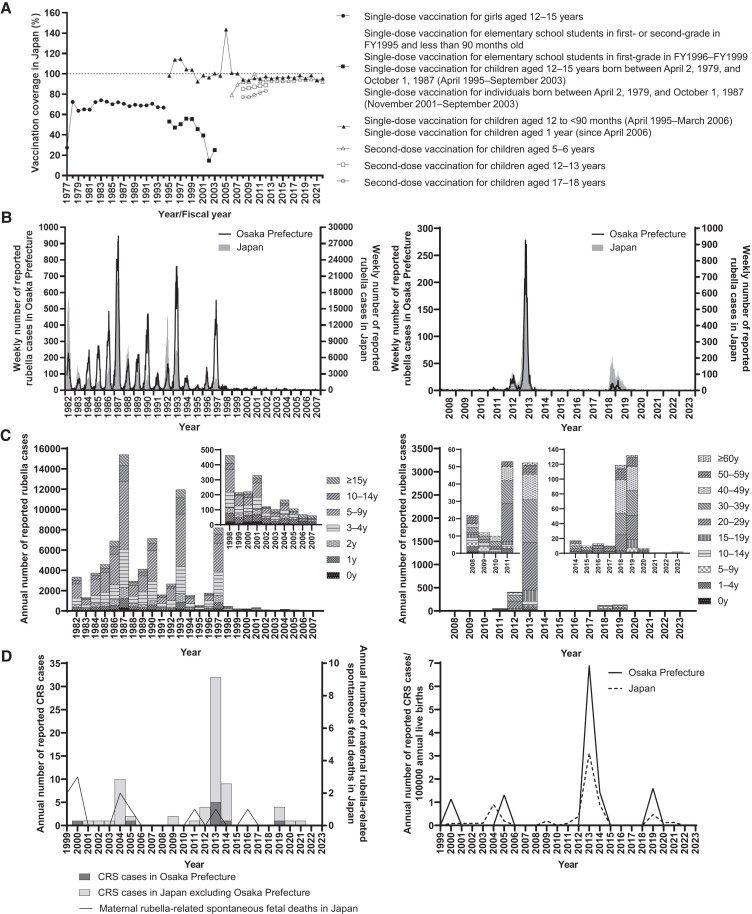
Immunization strategies and epidemiology of rubella, congenital rubella syndrome (CRS), and maternal rubella-related spontaneous fetal death. *A*, The vaccination strategy and reported vaccination coverage in Japan from 1977 to fiscal year (FY) 2022. *B*, The number of reported rubella cases in Osaka prefecture and Japan from 1982 to 2023. *C*, The age distribution of reported rubella cases in Osaka prefecture from 1982 to 2023. *D*, The number of reported CRS cases in Osaka prefecture and Japan and the number of maternal rubella-related spontaneous fetal deaths in Japan from 1999 to 2023. The vaccination coverage and epidemiologic data in Japan were obtained from the data published by the Ministry of Health and Welfare, Ministry of Health, Labour and Welfare, and the National Institute of Infectious Diseases in Japan [7, 8, 16]. Since 1997, vaccination coverage was calculated for each FY. The Japanese FY runs from 1 April to 31 March. As the target population is the population newly eligible for immunization in each year and the immunized population is the number of persons immunized among the entire population eligible for immunization in each year, the vaccination coverage may exceed 100%. National data of CRS were sourced from publicly available information of the National Institute of Infectious Diseases in Japan [28]. National data on number of births and maternal rubella-related spontaneous fetal deaths were sourced from Vital Statistics of Japan [29]. The left and right y-axes in panels *B* represent the data in Osaka prefecture and Japan, respectively. The data set on which this figure is based is provided in Supplementary Tables 1 to 7.

The timeline of vaccination strategy and coverage in Japan is shown in Figure 1*A*. In addition, the number of rubella cases in Osaka prefecture and Japan from 1982 to 2023 is presented in Figure 1*B* and 1*C*. From 1982 to 1994, when routine immunization targeted primarily adolescent girls, the average annual number of rubella cases was 5173 (range, 1317–15 397) in Osaka prefecture and 134 894 (range, 35 883–411 772) in Japan. In 1995, the target of routine immunization was changed to include children. This change reduced the average annual number of rubella cases to 1110 (range, 102–8193) in Osaka prefecture and 12 156 (range, 895–47 599) in Japan during 1995 to 2005. After the introduction of 2-dose vaccination in 2006 and notifiable disease surveillance in 2008, the average annual number of rubella cases was 24 (range, 10–53) in Osaka prefecture and 226 (range, 87–378) in Japan during 2008 to 2011. However, in Osaka prefecture and Japan, the number of rubella cases increased to 408 and 2386 in 2012 and to 3192 and 14 344 in 2013, respectively. Following this epidemic, the average annual number of rubella cases decreased to 13 (range, 10–17) in Osaka prefecture and 175 (range, 91–319) in Japan during 2014 to 2017. Yet again, in Osaka prefecture and Japan, the number of rubella cases increased to 119 and 2941 in 2018 and to 132 and 2298 in 2019. Thereafter, the number of rubella cases was only ≤2 and ≤15 per year in 2021 to 2023 in Osaka prefecture and Japan. The epidemic trend in Osaka prefecture mirrored that in Japan. The diagnosis rate based on laboratory tests increased from 69.0% (2585/3747) in 2008 to 2017 to 96.9% (254/262) in 2018 to 2023. Although RuV has not been detected since 2021, rubella cases have been reported per the results of an anti–RuV-specific IgM antibody test.

The number of CRS cases and maternal rubella-related spontaneous fetal deaths is shown in Figure 1*D*. Since 1999, a total of 9 and 70 patients with CRS were reported in Osaka prefecture and Japan, respectively [28]. Moreover, 11 cases of maternal rubella-related spontaneous fetal deaths were reported in Japan [29]. In 2013, the incidence rate of CRS per 100 000 live births in Osaka prefecture and Japan was 6.9 and 3.1.

#### Age Distribution of Rubella Cases

The age distribution of rubella cases from 1982 to 2023 in Osaka prefecture is shown in Figure 1*C*. From 1982 to 1994, the proportion of children aged <10 years ranged from 79.8% to 84.6%. This proportion began to decrease in 2003, reaching 57.4% and 59.0% in 2006 and 2007, respectively. After 2008, the average proportions of children aged <10 years and individuals aged ≥20 years were 12.0% (range, 0%–38.5%) and 78.6% (range, 50.0%–100%). The average number of rubella cases among children aged 1 to 9 years decreased from 4127 (range, 1047–12 429) during 1982 to 1994 to 747 (range, 30–6458) during 1995 to 2007. After the introduction of the childhood immunization program in 1995, a decrease in the number of rubella cases was observed in all age groups, including those not targeted for vaccination. During 2008 to 2023, the total number of rubella cases among individuals aged <20 years and those aged ≥20 years was 580 and 3429.

#### Characteristics of Patients With Rubella and CRS in Osaka Prefecture

The characteristics of rubella cases reported in Osaka prefecture are detailed in Table 1. Most cases presented with typical symptoms such as rash (99.6%), fever (88.2%), and lymphadenopathy (71.1%). Other clinical manifestations included arthritis/arthralgia (18.7%), conjunctivitis (6.5%), cough (2.0%), rhinitis (1.4%), thrombocytopenic purpura (0.4%), liver dysfunction (0.2%), and encephalitis (0.05%). Notably, the incidence of fever, arthritis/arthralgia, and conjunctivitis was significantly higher in individuals aged ≥20 years as compared with those aged <20 years. Regarding CRS cases (n = 9), 1 (11.1%) mother had a history of receiving a single-dose vaccination. In addition, 2 (22.2%) mothers had no history of rubella-like symptoms.

**Table 1.** Characteristics of Rubella Patients Reported (2008–2023)

	Age, y			
Characteristic	<20	≥20	Total	*P* Value^a^
Patients	580 (14.5)	3429 (85.5)	4009 (100.0)	…
Males	327 (56.4)	2596 (75.7)	2923 (72.9)	<.001
Clinical symptom				
Rash	578 (99.7)	3414 (99.6)	3992 (99.6)	>.99
Fever	483 (83.3)	3052 (89.0)	3535 (88.2)	<.001
Lymphadenopathy	433 (74.7)	2418 (70.5)	2851 (71.1)	.042
Arthritis/arthralgia	44 (7.6)	704 (20.5)	748 (18.7)	<.001
Conjunctivitis	17 (2.9)	243 (7.1)	260 (6.5)	<.001
Cough	7 (1.2)	74 (2.2)	81 (2.0)	.152
Rhinitis	7 (1.2)	50 (1.5)	57 (1.4)	.849
Thrombocytopenic purpura	2 (0.3)	15 (0.4)	17 (0.4)	>.99
Liver dysfunction	1 (0.2)	9 (0.3)	10 (0.2)	>.99
Encephalitis	1 (0.2)	1 (0.03)	2 (0.05)	.998

Data are presented as No. (%).

^a^Fisher exact test.

### Molecular Epidemiology of RuV

The sequences of the 739-nucleotide window region detected in Osaka prefecture from 1994 to 2020 were genetically characterized. These sequences were phylogenetically classified into genotypes 1E (n = 139), 2B (n = 61), 1J (n = 1), and 1D (n = 1).

#### Genotype 1E

**Figure 2.**
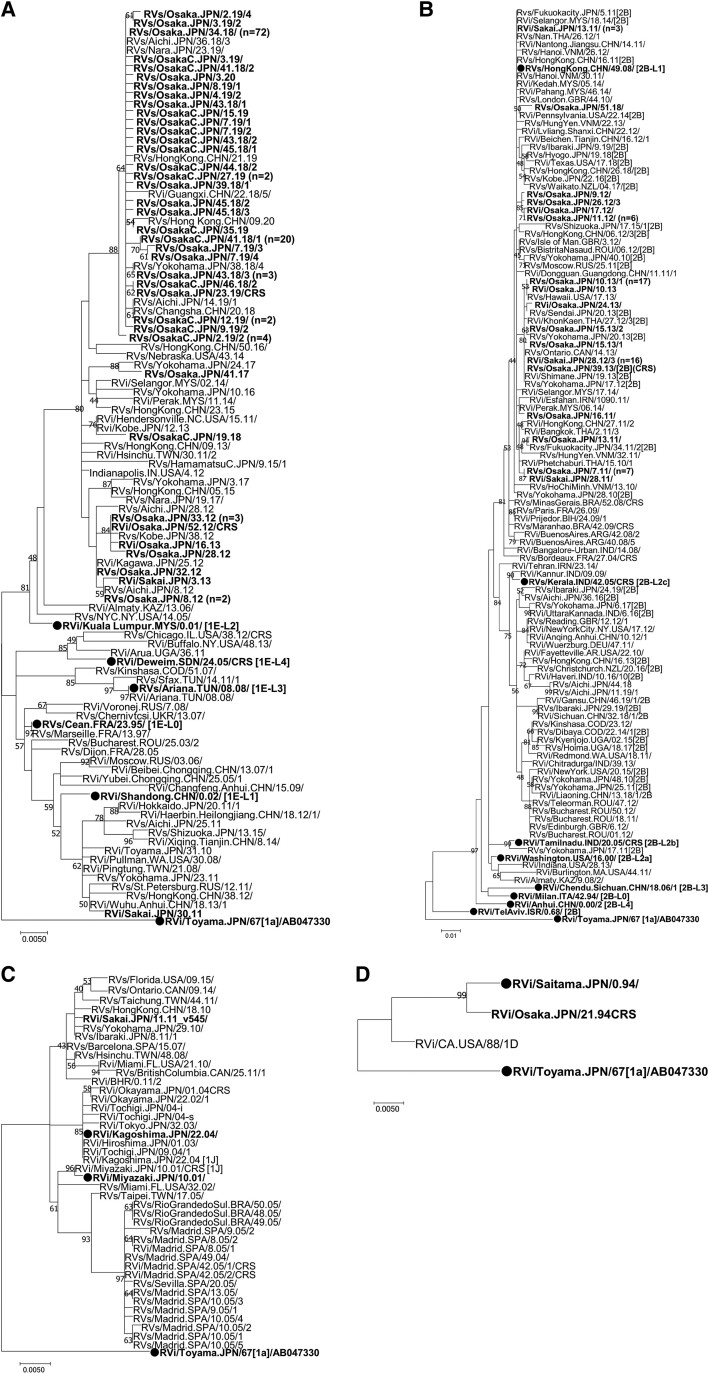
Maximum likelihood phylogram of the molecular window region within the *E1* gene of rubella virus: *A–D*, genotypes 1E, 2B, 1J, and 1D, respectively. A phylogenetic tree was constructed from sequences obtained during this study period, including previously reported sequences [25, 30, 31], sequences of the genotype reference strains [19], proposed candidate lineage reference strains [20], and representative strains detected in other countries and regions, as indicated in Supplementary Tables 8 to 13. The reliability of the tree at each branch node was assessed by the bootstrap method with 1000 replicates. Numbers at nodes represent bootstrap support values, given as a percentage of 1000 replicates (values <40 are omitted). The strains detected in Osaka prefecture are emphasized in bold. Genotype reference and proposed lineage reference strains are denoted by black circles. The genotype 1a strain (RVi/Toyama.JPN/67 [1a]) is included as an outgroup. In cases where there were multiple rubella viruses with the same sequence, only 1 representative strain is listed in the phylogenetic tree, with the number of strains sharing the same sequence shown in parentheses.

Genotype 1E strains were detected from rubella (n = 137) and CRS (n = 2) cases in 2011 (n = 1), 2012 (n = 7), 2013 (n = 3), 2017 (n = 1), 2018 (n = 59), 2019 (n = 67), and 2020 (n = 1). The genotype 1E strains detected in 2017 and 2020 were imported from Indonesia [30] and the Philippines, respectively. The genotype 1E strains identified in Osaka prefecture were further subdivided into 1E–lineage 1 (detected in 2011, n = 1) and 1E–lineage 2 (n = 138; Figure 2*A*). The 1E–lineage 2 strains detected in 2012 to 2013 and 2018 to 2019 formed distinct clusters, which were supported by 80% of the bootstrap values. All genotype 1E–lineage 2 strains detected in Osaka prefecture after week 34 in 2018 shared identical or very close genetic backgrounds with one another and with strains detected in mainland China and Hong Kong in 2018 to 2019.

#### Genotype 2B

Genotype 2B strains were identified from rubella (n = 60) and CRS (n = 1) cases in 2011 (n = 12), 2012 (n = 11), 2013 (n = 37), and 2018 (n = 1). All genotype 2B strains found in Osaka prefecture were classified as 2B–lineage 1 (Figure 2*B*). The RuV strains showed close relationships with strains detected in mainland China, Hong Kong, Vietnam, and other countries.

#### Genotypes 1J and 1D

The genotype 1J strain was identified in a rubella case in 2011 (Figure 2*C*). This strain was closely related to strains found in Hong Kong and Japan in 2010 to 2011. The genotype 1D strain was detected in a CRS case in 1994, which was also found in Saitama prefecture in the same year (Figure 2*D*).

### Serologic Analysis of Patients With Acute Rubella

**Figure 3.**
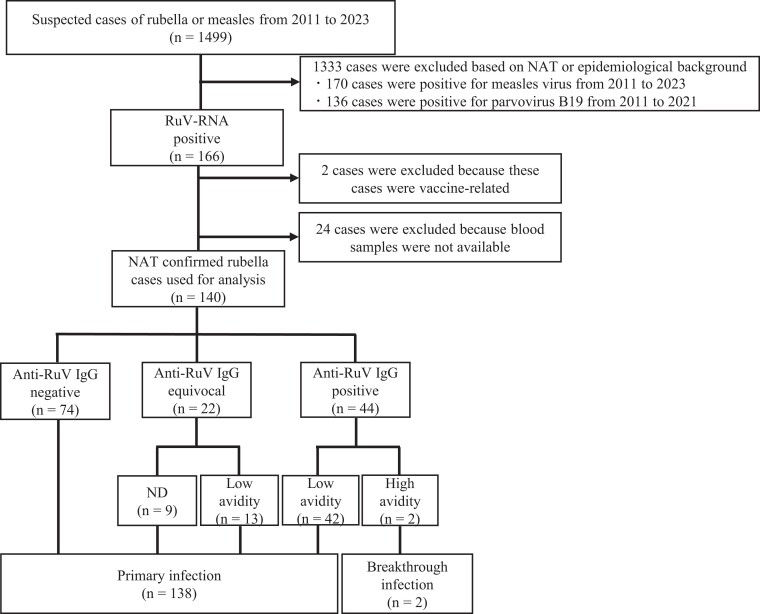
Differentiation between primary and breakthrough infections in rubella cases since 2011. Nucleic acid amplification test (NAT) was performed to detect rubella virus (RuV) and measles virus in suspected cases of rubella or measles. In addition, NAT was performed to detect parvovirus B19 in cases in which rubella or measles was excluded [34]. The titer and avidity of anti–RuV-specific IgG antibodies were measured in NAT-confirmed rubella cases by blood samples collected during the acute phase of infection. IgG, immunoglobulin G; ND, not determined (due to low antibody titers).

Measurement of avidity can distinguish between primary infections (including primary vaccine failure) and breakthrough infections [1, 32, 33]. To identify the host factors contributing to recent rubella epidemics, we analyzed the titer and avidity of anti–RuV-specific IgG antibodies in blood samples obtained from NAT-confirmed rubella cases (Figure 3). Of 1499 suspected cases of rubella or measles from 2011 to 2023, RuV RNA was detected in 166 cases. Of these, 140 cases were included in this analysis. The median age of these cases was 36 years (range, 0–62), and 81.4% (114/140) were male. The median time from rash onset to specimen collection was 1 day (range, 0–9). Of the 140 cases, 44 were positive for anti–RuV-specific IgG antibodies, 22 were equivocal, and 74 were negative. The avidity of anti–RuV-specific IgG antibodies was measured in 66 cases that tested positive or equivocal for these antibodies. Of the 66 cases, RAI was ≥60% in 2 cases and <40% in 55 cases, and in 9 cases, RAI could not be determined due to low antibody titers. The 2 cases with high-avidity antibodies were females aged 33 and 50 years in 2013 and 2018, respectively (ie, of the generation considered to have a history of single-dose vaccination). Both cases presented with fever and rash, and 1 case also had lymphadenopathy.

## DISCUSSION

**Figure 4.**
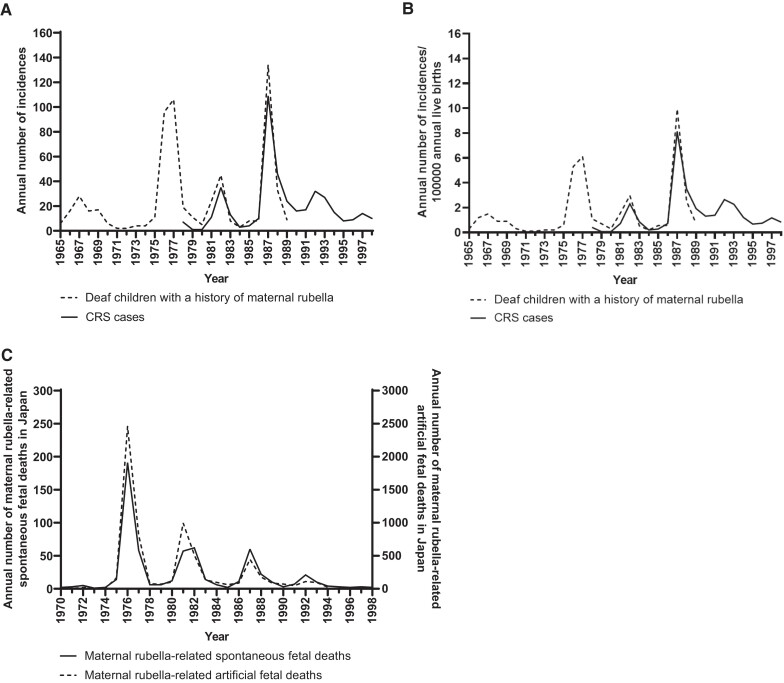
Annual incidence of congenital rubella syndrome (CRS) cases, deaf children, and maternal rubella-related spontaneous or artificial fetal deaths. Annual incidence of CRS cases and deaf children related to maternal rubella virus (RuV) infection: *A*, in Japan; *B*, per 100 000 live births in Japan. *C*, Annual incidence of maternal rubella-related spontaneous or artificial fetal deaths in Japan. The data on the annual incidence of CRS cases and deaf children with a history of maternal rubella were sourced from previous studies by Katow [35], Ueda et al [36], and Kadoya et al [37]. National data on maternal rubella-related spontaneous or artificial fetal deaths were sourced from Vital Statistics of Japan [29] and are provided in Supplementary Table 7.

Surveillance data showed that viral transmission persisted in the population even after the introduction of selective immunization. In addition, CRS cases and maternal rubella-related spontaneous or artificial fetal deaths were persistently reported (Figure 4) [29, 35–37]. Although the limited effectiveness of selective immunization has been noted [38], these data underscore the inadequacy of individual protection to control rubella and CRS. In April 1995, Japan switched to a universal approach [6], which resulted in a significant reduction in rubella cases, not only in the age groups targeted for routine immunization, but also in the nontargeted age groups. The universal approach may interrupt RuV transmission by reducing the infectious reservoir in children, thereby reducing the overall risk of infection and providing indirect protection to the unvaccinated population. However, rubella has reemerged as an adult infectious disease, albeit at a reduced epidemic scale. Although the disease burden of CRS may have been suppressed in the past by the large number of maternal rubella-related spontaneous or artificial fetal deaths (Figure 4), the incidence rate of CRS was comparable to that of the 1960s to 1980s [35, 37]. This would be attributed to the rubella epidemic in the generation of women of childbearing age in their families and an improved surveillance system. In addition, factors contributing to the highest incidence rate of CRS in Osaka prefecture in 2013 would be the highest incidence rate of rubella in Japan and active testing recommendations to hospitals and clinics.

Regarding the genotype of RuV, strains of genotypes 1D and 1J were identified in Osaka prefecture in 1994 and 2011, respectively. These genotypes were also found in other regions of Japan around the same periods. Interestingly, these specific genotypes have not been detected anywhere in Japan, including Osaka prefecture, in the past decade. This absence strongly suggests that the transmission of these RuV strains has been blocked, which is a significant accomplishment attributed to the implementation of vaccination program. In recent epidemics, almost all detected strains had identical or very similar genetic backgrounds to epidemic strains found in other countries, as documented in our previous reports [25, 31]. These results suggest that the influx of RuV from endemic countries is a contributing factor to the recent rubella epidemics.

**Figure 5.**
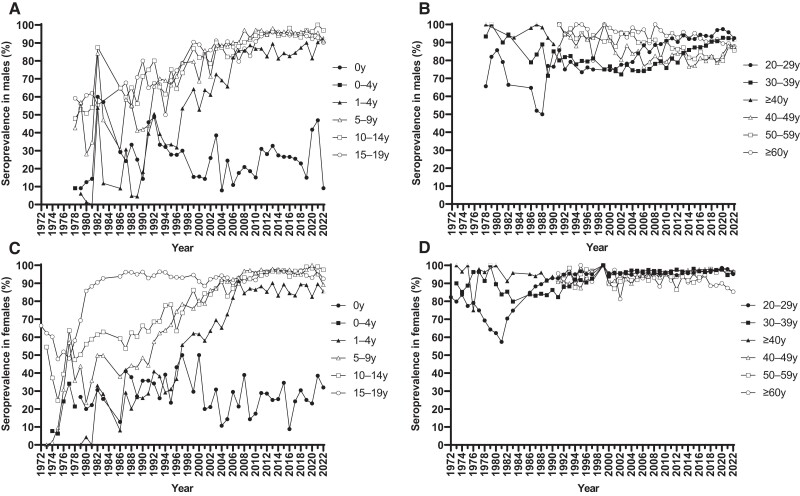
Age-specific seroprevalence against the rubella virus (RuV) from 1972 to 2022. The data are categorized into 4 groups by sex and age: *A*, males <20 years; *B*, males ≥20 years; *C*, females <20 years; *D*, females ≥20 years. In this study, a hemagglutination inhibition antibody titer ≥8-fold was interpreted as positive. The data for this analysis were sourced from the “Annual Report National Epidemiological Surveillance of Vaccine-Preventable Diseases” spanning 1972 to 2022 [39]. This serial cross-sectional serologic survey has been conducted almost annually, typically from July to September. Due to changes in age categories over time, the age distribution was summarized as follows (in years): 0, 1–4 (or 0–4), 5–9, 10–14, 15–19, 20–29, 30–39, 40–49, 50–59, and ≥60 (or ≥40). The data set used to create this figure is provided in Supplementary Tables 14 and 15.

In an effort to identify the host factors responsible for the recent rubella epidemics, we performed a serologic analysis of patients with rubella. The results showed that 98.6% (138/140) of rubella cases were confirmed as primary infections, even though the majority of the Japanese population has acquired immunity to RuV. The fact that the introduction of RCVs has led to a reduction in the number of rubella cases and that breakthrough infections were rare indicates the effectiveness of current vaccines against circulating RuV strains. However, the adult male population not targeted for routine immunization has been left susceptible to RuV, creating a high-risk situation for importation and indigenous circulation of RuV, especially in the densely populated regions. Our question is how this susceptible population was created. We retrieved data of seroprevalence from the National Epidemiological Surveillance of Vaccine Preventable Diseases spanning 1972 to 2022 [39], as summarized in Figure 5. Prior to the introduction of the vaccination program (1972–1976), seroprevalence among females aged 15 to 19 years was only 60.0% (3932/6552), despite repeated large rubella epidemics. The introduction of selective immunization in 1977 and universal immunization in 1995 led to a dramatic increase in seroprevalence in the target population. Furthermore, seroprevalence among children aged 1 to 4 years exceeded 80% in 2006 and has been maintained above this level since then. While the seroprevalence among males aged ≥40 years was generally >90% until the early 1990s, due to natural infection, it has gradually decreased among males in their 40s and 50s, reaching its lowest levels of 76.7% in 2014% and 79.8% in 2019, respectively. These results clearly illustrate that initiating childhood vaccination programs without considering the existence of susceptible populations might increase the number of susceptible adults and shift the disease to older groups, because the reduction and disappearance of rubella epidemics reduces the chance of infection and eliminates the opportunity to acquire immunity if the vaccine is not administered. Finally, although the major factors of recent epidemics have been discussed here, it is important to note that cases of breakthrough infection (1.4%, 2/140) and CRS (11.1%, 1/9) were identified in females who were considered to have a history of single-dose vaccination. Yet, none of the females with breakthrough infection or CRS were considered to have received a second-dose vaccination. Similarly, it has been reported that none of the mothers of patients with CRS associated with the 2012–2013 epidemic had a history of receiving a second-dose vaccination before pregnancy, although 11 mothers (24%, 11/45) had received a single-dose vaccination [40]. These facts suggest that single-dose vaccination cannot completely prevent CRS and breakthrough infection, indicating the need for second-dose vaccination. Therefore, continuation of the current 2-dose vaccination program would eliminate rubella and CRS, although the declined vaccination coverage during the COVID-19 epidemic should be improved.

Our study has several limitations. First, the surveillance system underwent changes during the study period, which makes it difficult to accurately compare before and after 2008. Second, our analysis could not include rubella cases without characteristic clinical symptoms. Therefore, our surveillance may underestimate cases of rubella and breakthrough infection. Third, although the positive predictive value of anti–RuV-specific IgM serology should be reduced by false positives in countries that have eliminated or nearly eliminated rubella, further confirmation of IgM-positive cases was not performed. Despite these limitations, our experience underscores the critical and evolving aspects of a vaccination program to eliminate and eradicate rubella.

## CONCLUSIONS

Epidemiologic analysis of rubella and CRS over 40 years has shown that selective immunization continues to cause infection transmission and severe outcomes, while universal immunization brings rubella under control. However, future epidemics and severe outcomes cannot be prevented unless the immunization gaps are closed. Also, CRS and breakthrough infections are not completely prevented by single-dose vaccination, indicating the need for second-dose vaccination. In addition, our experience would provide valuable lessons for the control of other infectious diseases with infectivity and transmission dynamics similar to RuV.

## Supplementary Data

Supplementary materials are available at *The Journal of Infectious Diseases* online (http://jid.oxfordjournals.org/). Supplementary materials consist of data provided by the author that are published to benefit the reader. The posted materials are not copyedited. The contents of all supplementary data are the sole responsibility of the authors. Questions or messages regarding errors should be addressed to the author.

## Supplementary Material

jiae402_Supplementary_Data

## Notes

***Acknowledgments.*** We appreciate the clinicians, government offices, and local public health centers in Osaka prefecture, Osaka city, and Sakai city for their collaboration in the surveillance of viral infectious diseases. We also thank the staffs of the Osaka Institute of Public Health (formerly the Osaka City Institute of Public Health and Environmental Sciences and the Osaka Prefectural Institute of Public Health) and the Sakai City Institute of Public Health for their longitudinal contribution. Finally, we thank Lennon P., working for Enago, for reviewing the English version.

***Author contributions*.** D. K.: conceptualization, formal analysis, investigation, data curation, visualization, project administration, writing–original draft, writing–review and editing, funding acquisition. T. Kurata: formal analysis, investigation, data curation, project administration, writing–review and editing, funding acquisition. Y. K.: formal analysis, investigation, data curation. T. M.: formal analysis, investigation, data curation. F. O.: formal analysis, investigation, data curation. T. Kase: formal analysis, investigation, writing–review and editing. J. K.: formal analysis, investigation, writing–review and editing. K. T.: formal analysis, investigation, writing–review and editing. K. I.: formal analysis, investigation, writing–review and editing, funding acquisition. K. M.: formal analysis, investigation, writing–review and editing, funding acquisition.

***Data availability*.** The data that support the findings of this study are available on request to the corresponding author.

***Declaration of artificial intelligence*.** During the preparation of this work, the authors did not use generative AI and AI-assisted technologies.

***Disclaimer*.** The study sponsors had no role in the analysis and interpretation of data, in the writing of the report, and in the decision to submit the article for publication.

***Financial support*.** This study was supported by the Osaka Prefectural Government; Osaka city; Sakai city; Japan Society for the Promotion of Science KAKENHI (JP26860453, JP18K17367, and JP21K10432 to D. K.); and the Research Program on Emerging and Re-emerging Infectious Diseases from the Japan Agency for Medical Research and Development (JP22fk0108628).
